# On Certain Skin Diseases

**Published:** 1908-02-15

**Authors:** T. D. Savill

**Affiliations:** Clinic at St. John's Hospital for Diseases of the Skin.


### ON CERTAIN SKIN DISEASES.

Notes from Dr. T. D. SAVILL'S Clinic at St. John's Hospital for Diseases of the Skin.

[*Specially reported for* The Hospital.]

Urticaria Pigmentosa (sometimes called Sangster's Disease).

This case (a boy aged eight) has been under treatment for some few years. Urticaria pigmentosa is a somewhat rare condition, which comes on early in life, usually during the first few months. It consists of a series of recurrent crops of nettle-rash, appearing on any part of the body, and leaving behind them pigmented stains. We do not always see the urticarial spots—they come and go; but the pigmented stains are always present, and are readily diagnosed when the condition is once seen. Sometimes rubbing the pigmented parts will raise a wheal. The stains might be mistaken for a purpura or purpuric erythema, but for the long history of recurrent crops and the early age of the patient. The only other disease it is apt to be mistaken for is Xeroderma pigmentosa, a still rarer disease, which also comes on in early life, but Xeroderma pigmentosa starts usually on the uncovered parts of the body (face and neck), has no wheals, and presents permanent stains, with atrophic patches. In Urticaria pigmentosa, most of the pigmented stains only last a few months, and then fade away, and the recurrent wheals are pathognomonic. Pathologically, the disease indicates two inherent hereditary conditions. One is a vascular or angio-neurotic factor, which shows itself in the associated nervousness, and in a tendency to the vascular changes frequently observed in these patients. There are also blood changes, allied to hæmophilia, which show themselves in the pigmented effusions. In ordinary urticaria, on the other hand, the exudation is simply serous.

There are three kinds of exudation met with in skin diseases. One is serous, such as occurs in urticaria, and in this condition the exudation dies away and leaves no stain behind; another is sero-sanguineous, which presents a certain amount of pigmentation, such as is seen, for instance, in erythema. Finally, there is a sanguineous exudation, such as occurs in purpura. Urticaria pigmentosa is attended by a sero-sanguineous exudation, and the cause of this, in my view, is (1) an inherent weakness in the vessel walls, and (2) an innate fluidity of the blood allied to hæmophilia. There are some who dispute this view of Urticaria pigmentosa, and modern histological methods have discovered in the more persistent lesions a mast-cell infiltration, but I am not satisfied that histological methods are the best for studying the pathology of a generalised skin disease. Histology is useful in the localised forms of skin disease, and is indeed an essential aid to the diagnosis and treatment of the skin in such cases. But when a skin disease is part of a constitutional condition or malady you cannot study it merely by histology, any more than you can study malaria by making sections of the spleen.

Treatment.

This child has improved very much under prolonged treatment. Lime is a most important constituent of the blood, and it is generally found to be deficient when the blood exudes from the vessels too readily. During the outbreaks of urticaria, this case is treated with what I call "Mistura ricini calcis." Its composition is as follows:—

| | |
|---|---|
| Castor oil ... .. ... ... ... | ℥j. |
| Powdered gum acacia ... ... ... | ʒij. |
| Syrup of orange ... ... ... ... | ʒij. |
| Saccharated solution of lime ... ... | ʒij. |
| Lime water, up to ... ... ... ... | ℥ij. |

Dose, one teaspoonful.

At the beginning of an outbreak the patient has also had (fairly constantly) Calcium chloride gr. v.; Ammonium bromide gr. v.; Liquor arsenicalis m. iij. Twice a day after meals. The calcium is given to increase the coagulability of the blood; the bromide of ammonium to allay the irritability of the angio-neurotic system; and the liquor arsenicalis as a nerve tonic. No local applications to the skin are any good.

Organic acids diminish the coagulability of the blood very much—citric and acetic acids particularly—because they dissolve the calcium out of the blood; therefore acids should be avoided in the treatment of such cases.

There is another point about this interesting case; the boy contracted ringworm at school. At first I was compelled to give him the usual old-fashioned lotions and ointments, because he got into such terrible panics at the idea of having *x*-rays. These cases are always very nervous and hysterical. It was found, as we expected, that his skin could not stand even the mildest applications. Later Dr. Agnes Savill persuaded him to let her apply the *x*-ray tube; it destroyed the ringworm very promptly, had not any effect on the skin, and improved the spots of urticaria pigmentosa in that situation.

### The Rashes of Scarlet Fever and Erythema Scarlatiniforme.

The differentiation between the rashes of Scarlatina and Erythema scarlatiniforme is extremely important, and may be set down in tabular form.

| Scarlatina. | Erythema Scarlatiniforme. |
|---|---|
| 1. Temperature—Sudden advent, high. | 1. Not much pyrexia or headache. |
| 2. Throat involved. | 2. Throat not involved. |
| 3. Rash diffused, non-marginated, with punctate element. | 3. Rash tends to be localised, marginated, non-punctate, duration considerable. |
| 4. Tongue strawberry, second or third day. | 4. Nothing characteristic. |
| 5. Exfoliation general — Desquamation later on. | 5. Early desquamation. |

The diagnosis between these two conditions is important, and sometimes very difficult; but in typical cases of scarlatina we have four points of differentiation:—

1. *Sudden* advent of high temperature. There are three fevers which show this phenomenon. We should always suspect with a sudden high temperature one of three diseases:—scarlet fever, erysipelas, or small-pox. The onset may be attended by vomiting.

2. The next thing to appear is a scarlet redness of the throat, which does not affect the larynx, but only the fauces and the tonsils.

3. The rash appears on the second day, and may be so evanescent that it disappears at the end of thirty-six hours; usually it lasts three or four days. It consists of a generalised erythema with punctate crimson spots or papules. It appears first on the neck and chest.

4. The strawberry tongue appears at the end of the second or third day.

In Erythema scarlatiniforme, the pyrexia does not often go above 100° or 100.5°. The patient does not usually complain of headache or of any other symptoms of a constitutional disturbance. There is no strawberry tongue, but the rash is present in both, and it is sometimes very difficult to diagnose between the two. The rash of Erythema scarlatiniforme is generally more or less diffused, with a tendency to be limited or to predominate on the flexor aspects, and on the abdomen. It is often patchy, but symmetrical. The punctate element is usually, but not always, wanting. The eruption lasts from one to four weeks, much longer than that of scarlet fever. Exfoliation generally follows. Recurrence is frequent. Both eruptions are due to a toxin that tends to poison the epithelial structures of the body, that is to say, the skin and the kidneys. In scarlatina and small-pox the ends of the extremities are for some reason or other affected very constantly, and at a later stage than the other parts. In scarlet fever patients you always look at the feet to ascertain if they are free from infection, because they are the last to exfoliate. The peeling here is useful to the physician, because it shows the termination of the disease.

### The Rash of Scabies.

Scabies is usually a papular, sometimes a multiform eruption, especially in children. The rash of scabies attacks the flexor surfaces of the wrists, the clefts of the fingers, the axillæ, the fronts of the arms, the groins, and the abdomen. These are the characteristic positions of scabies. The essential features are itching, and the presence and the distribution of the papules. By these points one can differentiate it from idiopathic prurigo. I regard many eruptions (scabies, lichen urticatus, prurigo senilis, etc.), which present itching associated with papules, as coming under one head, and belonging to one great clinical and pathological group—Prurigo. It is by the characteristic distribution of the papules that scabies is differentiated clinically from all other forms of prurigo, and, of course, histologically by the presence of the acarus scabiei. But the insect is often very difficult and always tedious to find.

